# Models for predicting the risk of bloodstream infections associated with peripherally inserted central venous catheters: A scoping review

**DOI:** 10.1371/journal.pone.0333466

**Published:** 2025-10-06

**Authors:** Langping Cao, Yingxiang Tao, Shiqiang Lei, Shihua He, Yuxuan Peng, Sailin Liu, Haoran Chen, Jin Zhou, Yanhui Liu

**Affiliations:** 1 Teaching and Research Section of Clinical Nursing, Xiangya Hospital, Central South University, Changsha, Hunan, China; 2 Department of Neurosurgery, Xiangya Hospital, Central South University, Changsha, Hunan, China; Ataturk University Faculty of Medicine, TÜRKIYE

## Abstract

**Background:**

Peripherally inserted central venous catheters (PICC) associated bloodstream infections is a type of central line-associated bloodstream infection (CLABSI), the purpose of this scoping review was to analyse and summarize the risk prediction models for PICC-CLABSI to provide insights for clinical nursing practice.

**Methods:**

A scoping review was conducted from six bibliographic databases including CNKI, Wanfang Database, VIP Chinese Journal Database, PubMed, Embase, and Web of Science, from inception to November 5, 2024. Screening was performed and relevant data was extracted independently by two researchers, the risk of bias was assessed using the Prediction model Risk Of Bias Assessment Tool (PROBAST).

**Results:**

Eight studies met the inclusion criteria, which included two score models, six nomograms, and one ELM model. The included studies exhibited a high risk of bias, mainly due to methodological heterogeneity. Four models underwent external validation, two were assessed for goodness of fit. The most frequently identified predictors including catheter indwelling time, maintenance cycle/frequency, multilumen catheters, PICC parenteral nutrition, diabetes, and malignant tumors.

**Conclusion:**

The risk prediction models for PICC-CLABSI demonstrated strong predictive performance. Future research should carefully address all elements of PROBAST framework during study design phase. This will facilitate both internal and external validation, as well as differentiation, calibration, and evaluation of clinical practicality. The ultimate objective is to develop a PICC-CLABSI risk prediction model that exhibits low bias risk, robust predictive performance, and high clinical applicability.

## 1. Background

Peripherally inserted central venous catheters (PICC) associated bloodstream infections is a type of central line-associated bloodstream infection (CLABSI), onset 48 hours after the insertion of PICC at least. Studies have shown that the incidence rate of PICC-CLABSI ranges from 1.4% to 16.84% [1–4], which significantly affects patient treatment and prognosis, leading to prolonged hospitalization, higher readmission rates, increased medical costs, and even mortality. The literature reports a mortality rate of 10.26% for bloodstream infections related to PICC [5]. Those highlight the urgent need for effective strategies to prevent and manage PICC-CLABSI. Several factors contribute to the occurrence of PICC-related bloodstream infections, such as patient characteristics, underlying diseases, treatment methods, and catheter-related factors [6,7]. By identifying key risk factors and implementing evidence-based interventions, healthcare providers can improve patient outcomes and minimize the negative impact on recovery. In China, various departments and nursing organizations have publilshed guidelines, such as the “Technical operation standards for venous therapy nursing” [8] and the “Expert consensus on the maintenance of clinical venous catheters” [9]. In 2021, National Health Commission of the People's Republic of China has issued the annual nursing quality control goal, which is aimed at reducing the incidence of intravascular catheter-related bloodstream infections (CRBSI) [10]. To address high-risk factors and critical aspects of CLABSI, the National Nursing Management Professional Medical Quality Control Center plans to launch the “Quality control kit for preventing intravascular catheter-related blood flow infections (recommended version)” in 2023 [11]. Which provide standardized procedures and recommendations for the prevention and management of PICC-CRBSI. A risk prediction model is a statistical tool designed to estimate the probability of a specific outcome event occurring in a population with defined characteristics. Moons et al. outlined a comprehensive framework for developing and evaluating predictive models, including model development, internal validation, external validation, model updates, and impact studies [12]. Currently, researchers globally have explored the construction and efficacy of risk prediction models for PICC-related bloodstream infections. However, the quality of these studies varies significantly. This scoping review aims to summarize, analyze, and compare relevant research, with the objective of providing valuable insights that can inform and enhance clinical practice.

## 2. Materials and methods

### Identification of research question

This scoping review aims to address the following questions: (1) What types of predictive models are currently available for predicting the risk of PICC-CRBSI? (2) Which predictive factors are most frequently incorporated into these risk prediction models? (3) What modeling methods are currently used for PICC-CRBSI risk prediction, and how effective are they? (4) What are the limitations of existing studies on PICC-CRBSI risk prediction models, and what directions do these suggest for future research?

### Search strategies and eligibility criteria

A comprehensive literature search was conducted across multiple databases, including the China National Knowledge Infrastructure, Wanfang Database, VIP Chinese Journal Database, PubMed, Embase, and Web of Science. The search period covered from the inception of these databases up to November 5, 2024. Both subject headings and free-text terms were used in the retrieval process. For example, in PubMed, the search formula for the English database was: (vascular access devices [MeSH Terms] OR peripherally inserted central catheter [Title/Abstract] OR central venous catheters [MeSH Terms] OR PICC [Title/Abstract] AND (catheter-related infect [Title/Abstract] OR CRBSI [Title/Abstract] OR CLABSI* [Title/Abstract] OR bloodstream infect* [Title/Abstract] OR blood poison* [Title/Abstract])** AND (risk predict [Title/Abstract] OR risk score [Title/Abstract] OR risk assess [Title/Abstract] OR score [Title/Abstract] OR prognos* [Title/Abstract] OR nomogram model [Title/Abstract])** [13]. Details of eligibility criteria were included in Table 1.

**Table 1:** Scoping review eligibility criteria.

Inclusion criteria	Exclusion criteria
Aged ≥ 18 years	Studies including patients with other types of central venous catheterization
Received PICC placement	Duplicate publication
Study must focus on the development or validation of a risk model for PICC-CRBSI	Studiesfocused solely on risk factors without model development
The methods for model construction or validation must be clearly described	Literature from which the full text cannot be obtained
The literature must be published in Chinese or English.	Conference abstracts and review articles

### Data extraction

Two researchers (the first and third authors) independently conducted the literature screening according to the predefined inclusion and exclusion criteria, and extracted and integrated the relevant data. Any disagreements were resolved by a third reviewer (Yanhui Liu). Extracted data included: publication year, country, research design, research type, subject, sample size, incidence rate, modeling methods, candidate variables, variable screening methods, model presentation, model validation status, missing data, model predictive factors, and predictive performance.

### Bias risk and applicability evaluation

Two researchers (the first and third authors) evaluated the quality of the included literature using the Prediction Model Risk of Bias Assessment Tool (PROBAST) [14]. The bias risk assessment of predictive models using this tool covers four domains: research object, predictive factors, outcomes, and statistical analysis. Additionally, the assessment of adaptability focuses on three domains: research object, predictive factors, and outcomes. The risk of bias and concerns regarding applicability in each domain were rated as high, low, or unclear.

## 3. Results

**Fig 1:**
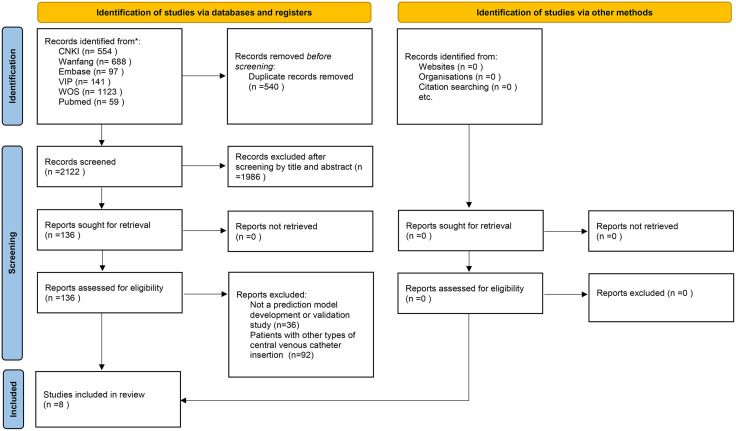
Flow_diagram.

After an initial search, we retrieved 2,662 articles, including 554 from the China National Knowledge Infrastructure, 688 from the Wanfang Database, 141 from the VIP Chinese Journal Database, 59 from PubMed, 97 from Embase, and 1123 from the Web of Science. Upon removing duplicates, reviewing titles and abstracts, and assessing the full texts, 8 articles were ultimately included (Fig 1).

### Characteristics of selected studies

Eight studies [15–21] were included in this study. Among these, one was a multicenter prospective cohort study [15], while the remaining seven were retrospective studies [16–21]. Six studies conducted both model development and validation [15,17,19–21], one study focused exclusively on model development [16], and another solely on model validation [18]. In terms of patient populations, one study specifically involved hospitalized medical adults [15], four were applicable to tumor patients [17,19,20,22] and three were targeted to hospitalized adults [16,18,21]. The basic characteristics of the included studies are detailed in Table 2.

**Table 2:** Characteristics of the included studies (n = 8).

Include studies	Publication year	Country	Research design	Study type	Subject	Sample size	CLABSI incidence rate (%)
Erica et al [15]	2017	America	Multi center prospective cohort study	Development and validation	Hospitalized medical adults	23088	1.10
Tang QY et al [16]	2020	China	Retrospective study	Development	Hospitalized adults	931	6.80
Tao Y et al [17]	2022	China	Retrospective study	Development and validation	Tumor patients	11901	0.39
Hirotaka et al [18]	2023	Japan	Retrospective study	Validation	Hospitalized adults	1459	6.10
Hao QY et al [19]	2023	China	Retrospective study	Development and validation	Lung cancer chemotherapy patients	701	10.13
Guo J et al [20]	2024	China	Retrospective study	Development and validation	Elderly patients undergoing chemotherapy for acute leukemia	568	8.1
Li W et al [21]	2024	China	Retrospective study	Development and validation	Hospitalized adults	505	14.85
Yu QQ et al [22]	2024	China	Retrospective study	Development and validation	Tumor patients	1146	0.28

### Assessment of literature bias risk and applicability

The PROBAST evaluation for bias risk, covering four aspects of model development—study population, predictive factors, outcomes, and analysis—indicated a higher risk of bias in the predictive factors and analysis sections. This elevated risk may be attributed to the selection of statistical methods for predictive factors and the consideration of complex statistical issues during model development. The results of the evaluation are summarized in Table 3.

**Table 3:** Evaluation of bias risk and applicability of included studies.

Include studies	Risk of bias				Applicability			Overall	
**Research object**	**Predictor**	**Outcome**	**Analysis**	**Research object**	**Predictor**	**Outcome**	**Bias**	**Applicability**
Erica et al [15]	+	+	+	+	+	+	+	+	+
Tang QY et al [16]	-	+	+	+	+	+	+	-	+
Tao Y et al [17]	-	?	+	+	+	+	+	-	+
Hirotaka et al [18]	+	?	+	+	+	+	+	-	+
Hao QY et al [19]	+	+	+	+	+	+	+	+	+
Guo J et al [20]	+	-	+	+	+	+	+	-	+
Li W et al [21]	+	?	-	-	+	+	+	-	+
Yu QQ et al [22]	-	+	+	-	+	+	+	-	+

Note: + low risk, ?unclear, -high risk.

### Construction and validation of the model

#### Basic information on model construction.

The study population for this model predominantly included elderly individuals, cancer patients, and hospitalized patients, with sample sizes ranging from 505 to 23,088. The modeling techniques utilized were Cox regression and logistic regression, involving between 5 and 24 candidate variables and 4–8 predictive factors in the analysis. The model was illustrated using scoring scales and line graphs, with both internal and external validations, see Table 4.

**Table 4:** Construction and validation of risk prediction model (n = 8).

Include studies	Modeling method	Candidate variables	Variable selection method	Model predictive factors	Model presentation method	Missing Data
Erica et al [15]	Cox regression	24	Cox regression	6:Hematological cancer, CLABSI within 3 months of PICC insertion, multilumen PICC, solid cancers with ongoing chemotherapy, receipt of total parenteral nutrition through the PICC, presence of another central venous catheter (CVC) at the time of PICC placement	Michigan PICC-CLABSI (MPC) score	Multiple imputation
Tang QY et al [16]	Logistic regression	12	X^2^test, t test, logistic regression	8:Diabetes mellitus, malignant tumor, hematopathy, parenteral nutrition, double lumen, additional devices, ICU stay, and the time of indwelling catheter	Nomogram	——
Tao Y et al [17]	Logistic regression	14	Logistic regression	4:Dermatitis, catheter-related thrombosis, local infection and exudation during PICC retention	Nomogram	——
Hirotaka et al [18]	——	——	——	6:Hematological cancer, CLABSI within 3 months of PICC insertion, multilumen PICC, solid cancers with ongoing chemotherapy, receipt of total parenteral nutrition through the PICC, presence of another central venous catheter (CVC) at the time of PICC placement	Michigan PICC-CLABSI (MPC) score	——
Hao QY et al [19]	Logistic regression	16	Logistic regression	7:Diabetes, the number of chemotherapy ≥ 5, history of hospitalization in intensive care unit, prolonged catheter maintenance time, catheter movement, catheter retention time ≥ 30 d, and the number of punctures ≥ 2	Nomogram	——
Guo J et al [20]	Logistic regression	5	X^2^ test, logistic regression	4:Chemotherapy frequency, single catheterization puncture frequency, whether catheterization maintenance frequency was standardized, and catheterization retention time	Nomogram	——
Li W et al [21]	Logistic regression	13	LASSO, Logistic regression	7:age > 60 years, catheter movement, catheter maintenance cycle >7 days, direct insertion, poor immune function, complications, body temperature ≥37.2°C before PICC placement	Nomogram	——
Yu QQ et al [22]	Logistic regressionand extreme learning Machine (ELM)	17	X^2^ test, logistic regression	6:Diabetes history, number of chemotherapy sessions, maintenance cycle, maintenance address, white blood cell count and albumin	Nomogram, ELM prediction model	——

Note: LASSO (the least absolute shrinkage and selection operator regression, LASSO)

#### Model prediction content.

The results were summarized and categorized. Ultimately, the predictors were classified into five categories: general information, catheter-related factors, disease-related factors, treatment factors, and other factors. The most frequently observed predictors included catheter retention time, maintenance cycle/frequency, multilumen, PICC parenteral nutrition, diabetes, and malignant tumors. The classification of model predictive factors is shown in Table 5.

**Table 5:** Classification of model predictive factors.

Items		Number of models included	Items		Number of models included
General Information	Age	1	Disease related factor	Dermatitis	1
Catheter related factors	Multilumen PICC	3		Infections	1
	Attachment	1		Solid cancers with ongoing chemotherapy	2
	Catheterization retention time	4		Immune function	1
	Catheter movement	2		Body temperature before PICC placement	1
	Exudation	1		Complications	1
	Maintenance cycle/frequency	3		Hematological disease	1
	Number of punctures	2		Malignant tumor	3
	Presence of another central venous catheter (CVC) at the time of PICC placement	2		Diabetes	3
				White blood cell count	1
				Albumin	1
	Direct insertion	1	Therapeutic factors	Receipt of total parenteral nutrition through the PICC	3
	CLABSI within 3 months of PICC insertion	2		Chemotherapy frequency	3
	Catheter-related thrombosis	1	Other factors	ICU hospitalization history	2
				Maintenance address	1

#### Model validation and performance.

**Fig 2:**
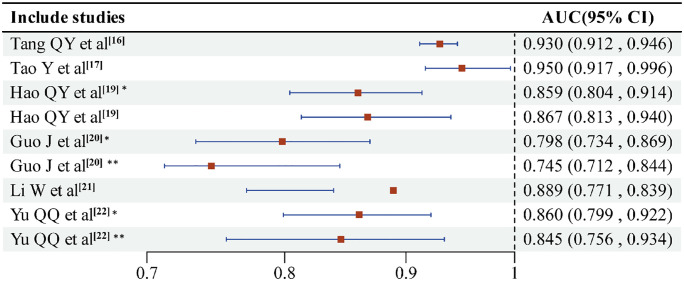
Forest plot of different model performances. The length of the blue line represents the width of the confidence interval, the red square represents the area under the curve (AUC) of the model, * corresponds to the development set of the model, and ** represents the validation set.

Among the eight models evaluated, four models [15–17,21] underwent internal validation, while the other four models were externally validated [18–20,22]. In terms of model performance, two models [19,22] employed the Hosmer-Lemeshow goodness-of-fit test, which indicated a good fit with X² = 8.905, P = 0.350 for the modeling set, and X² = 8.693, P = 0.365 for the validation set. Six models [15,16,19,22] reported the area under the curve (AUC) of the receiver operating characteristic curve, and two models [16,17] calculated the C-index to demonstrate their discriminative power. Additionally, five models [16–20] assessed model calibration. Two models [18,20] conducted decision curve analysis (DCA), with one noteworthy example by Hirotaka et al. [18] performing external validation on the Michigan Peripheral Insertion Center Catheter-Related Blood Flow Infection Rating Model [15] developed in 2017, demonstrating DCA’s superiority over the original rating scale. It is important to note that sensitivity and specificity were not reported for some models. In summary, the evidence indicates that each model is most reliable when applied to the patient group for which it was originally derived and externally validated: the Tang QY nomogram offers the strongest discrimination for general medical inpatients, the Hao QY nomogram performs best in lung-cancer patients undergoing chemotherapy, the Yu QQ nomogram and the ELM model are suitable for heterogeneous mixed-tumor cohorts, the Guo J nomogram is calibrated for immunocompromised elderly individuals with acute leukemia, and the large-scale MPC score provides a pragmatic baseline across diverse hospital settings. Together, these data underscore that model choice should be guided by alignment between derivation cohort and local case-mix, available clinical resources, and the extent of external validation in the intended population. Details of model validation and performance are presented in Table 6. Forest plot of models were drawed (Fig 2), two studies could not extract complete data, and no forest plot was generated [15,18].

**Table 6:** Model validation and performance.

Include studies	Validation	AUC^a^/C Index^b^	Calibration	DCA
Erica et al [15]	Internal validation	0.67 ~ 0.77^a^	——	——
Tang QY et al [16]	Internal validation	0.930^a^, 0.929^b^	Calibration curve (with an average absolute error of 0.017)	——
Tao Y et al [17]	Internal validation	0.825^b^	Calibration curve	——
Hirotaka et al [18]	External validation	——	The calibration slope is 1.16, *P* = 0.024, update the calibration slope to 1.02, *P* = 0.051	Superior to the original MPC score
Hao QY et al [19]	External validation	Development set: 0.859^a^ Validation set: 0.876^a^	Calibration curve: The incidence rate is basically consistent with the actual incidence rate; Hosmer-Lemeshow: Development set X^2^ = 8.905, *P* = 0.350; Validation set X^2^ = 8.693, *P* = 0.365	——
Guo J et al [20]	External validation	Development set: 0.798^a^ Validation set: 0.745^a^	Calibration curve	Has good clinical application efficacy
Li W et al [21]	Internal validation	0.889^a^	——	——
Yu QQ et al [22]	External validation	Nomogram: Development set: 0.860^a^ Validation set: 0.845^a^ ELM prediction model (Modeling set): R^2 ^= 0.823, mean squared error = 0.051	Hosmer-Lemeshow: Development set X^2^ =5.201, *P* = 0.736; Validation set X^2^ = 6.079, *P* = 0.531	——

## 4. Discussions

### Characteristics and validation needs of risk prediction models for PICC-CRBSI

Recent years have seen increasing scholarly interest in PICC catheter-related bloodstream infections, leading to a significant rise in studies aimed at their risk prediction [15–22]. This study systematically reviewed such research, both domestically and internationally, culminating in the inclusion of eight studies comprising six distinct risk prediction models. Only one of these was a multicenter prospective cohort study, with the others being retrospective, encompassing sample sizes from 505 to 23,088. The methodologies employed included Cox and logistic regression, with the number of candidate variables ranging from five to twenty-four, and predictive factors from four to eight. The models were presented using scoring scales and column charts. The AUC for six models ranged between 0.67 and 0.930 [15–17,19–21], while two models reported a C-index between 0.825 and 0.929 [16,17], indicating robust predictive performance. Key predictors in these models included catheter retention time, maintenance cycle/frequency, multilumen, PICC parenteral nutrition, diabetes, and malignancy. Clinically, it is essential to raise awareness and train medical personnel to promptly identify and address risk factors for PICC catheter-related bloodstream infections to prevent complications.

In terms of model construction, only one model utilized LASSO regression [21], and there are currently no reports in Chinese or English on using machine learning algorithms for developing risk prediction models for PICC catheter-related blood flow infections. LASSO regression plays a critical role in reducing dimensionality and selecting variables, effectively addressing multicollinearity when numerous influencing factors are involved [23]. Future developments in predictive modeling should consider employing LASSO regression and machine learning algorithms for feature selection, thereby enhancing predictive accuracy. Notably, 87.50% of the models included in this study were retrospective, highlighting the need for more multicenter, large-sample, prospective studies in the future.

Regarding model validation, four models [15–17,21] underwent internal validation, while four models [18–20,22] were externally validated. Internal validation is critical to prevent model overfitting, while external validation establishes a basis for model optimization and broader application. Upon completing model development, it is crucial to conduct both internal and external validation simultaneously and to perform multiple model comparisons to boost predictive accuracy and stability [24,25]. All models underwent discrimination testing, however, only two model [19,22] demonstrated goodness of fit. Five models [16–20] evaluated model calibration, and two models [18,20] assessed clinical utility, however, none of the studies addressed model sensitivity or specificity. For future construction of a risk prediction model for PICC catheter-related bloodstream infections, adherence to established clinical prediction model guidelines is recommended [24]. This should encompass evaluations of the model’s discrimination, calibration, goodness of fit, and clinical utility, as well as including specificity and sensitivity in research findings.

### The risk of bias in the PICC-CRBSI risk prediction model is significant and requires further optimization

PROBAST is an invaluable tool for evaluating the risk of bias and the applicability of predictive models [14]. This study’s findings reveal that while the included models showed good adaptability, they were associated with a high risk of bias, with only two models exhibiting a low risk. Among the eight studies included, only Erica et al. [15] employed a prospective approach, retrospective studies are especially prone to bias from existing outcomes, thereby increasing the risk of bias. Of the eight studies, only two conducted the Hosmer-Lemeshow goodness-of-fit test [19,22]. Furthermore, in the study by Guo J et al. [20], a limited number of five candidate variables and fewer than ten dependent events significantly increased the risk of model overfitting [26]. Some researchers increase bias risk by converting continuous variables into categorical ones in logistic regression modeling. For instance, Hirotaka et al. [18] used age as a binary variable, while Li W et al [21] transformed age, catheter retention time, and catheter insertion precursor temperature into binary variables. Furthermore, diagnostic criteria varied, Hao Qiyan et al. [19] adhered to the “Guidelines for the Prevention and Treatment of Endovascular Catheter-related Infections (2007)” [27], whereas Tang QY et al. [16] and Li W et al [21] followed the criteria published by the Centers for Disease Control and Prevention in the United States [28]. Additionally, some studies [17,18,21] failed to provide detailed data processing descriptions, indicating that comprehensive reporting is necessary during model development and validation processes.

### Impact of sample-size variability on model reliability and generalizability

Sample-size heterogeneity across the eight included studies influenced both the stability of individual prediction models and the precision of their reported performance metrics. Derivation cohorts with limited events-per-predictor parameter (EPP < 10)—observed in 6 of the 8 studies—are inherently prone to over-fitting, which can widen confidence intervals around discrimination (e.g., AUROC) and calibration statistics and thus raise concerns about internal validity [29]. Conversely, the two studies with EPP ≥ 10 benefited from reduced statistical noise and narrower intervals, although sheer size alone did not guarantee superior model accuracy. From a translational perspective, generalizability depends less on absolute cohort size than on population representativeness and the inclusion of external validation. In this review, studies spanning the full sample-size spectrum that incorporated external validation reported consistent performance between derivation and validation cohorts, indicating that adequate EPP and rigorous validation can offset potential drawbacks of modest cohort sizes. Therefore, while variable sample sizes introduce differential uncertainty, their impact on the collective evidence base is attenuated when methodological quality—particularly appropriate parameter-to-event ratios and robust external validation—is maintained [30].

## 5. Summary

In summary, this scoping review indicates that despite promising predictive performance, current risk prediction models for PICC-CRBSI suffer from substantial bias. Future research should focus on these priorities: 1) Performing external validation and enhancing existing prediction models; 2) Intensifying the investigation of risk factors associated with PICC-CRBSIin accordance with the PROBAST criteria, and developing a predictive model that minimizes bias risk while maximizing clinical applicability. The application of LASSO regression and machine learning methods may facilitate the integration of additional potential risk factors. It is vital todevelop tailored PICC-CRBSI risk prediction model for specific patient populations, such as cancer patients, ICU patients, outpatient chemotherapy patients, and home care patients, to improve the model’s specificity. Additionally, both internal and external validations should be conducted, accompanied by a comprehensive evaluation of the model’s predictive performance, applicability, stability, and other relevant attributes.

## Supporting information

S1 File Supplemental tables, figures. (DOCX)

S2 File Supplemental values used to build graphs. (XLSX)

S3 File PRISMA_2020_flow_diagram_new_SRs_v2. (DOCX)
