# Supplementary material for: Models for predicting the risk of bloodstream infections associated with peripherally inserted central venous catheters: A scoping review
Source: PLoS One. 2025 Oct 6;20(10):e0333466. doi: 10.1371/journal.pone.0333466 (PMC12500127; doi:10.1371/journal.pone.0333466)
Supplement: S1 File — (DOCX) [file pone.0333466.s001.docx]

Supplemental Figures and Tables

# Supplemental Figures


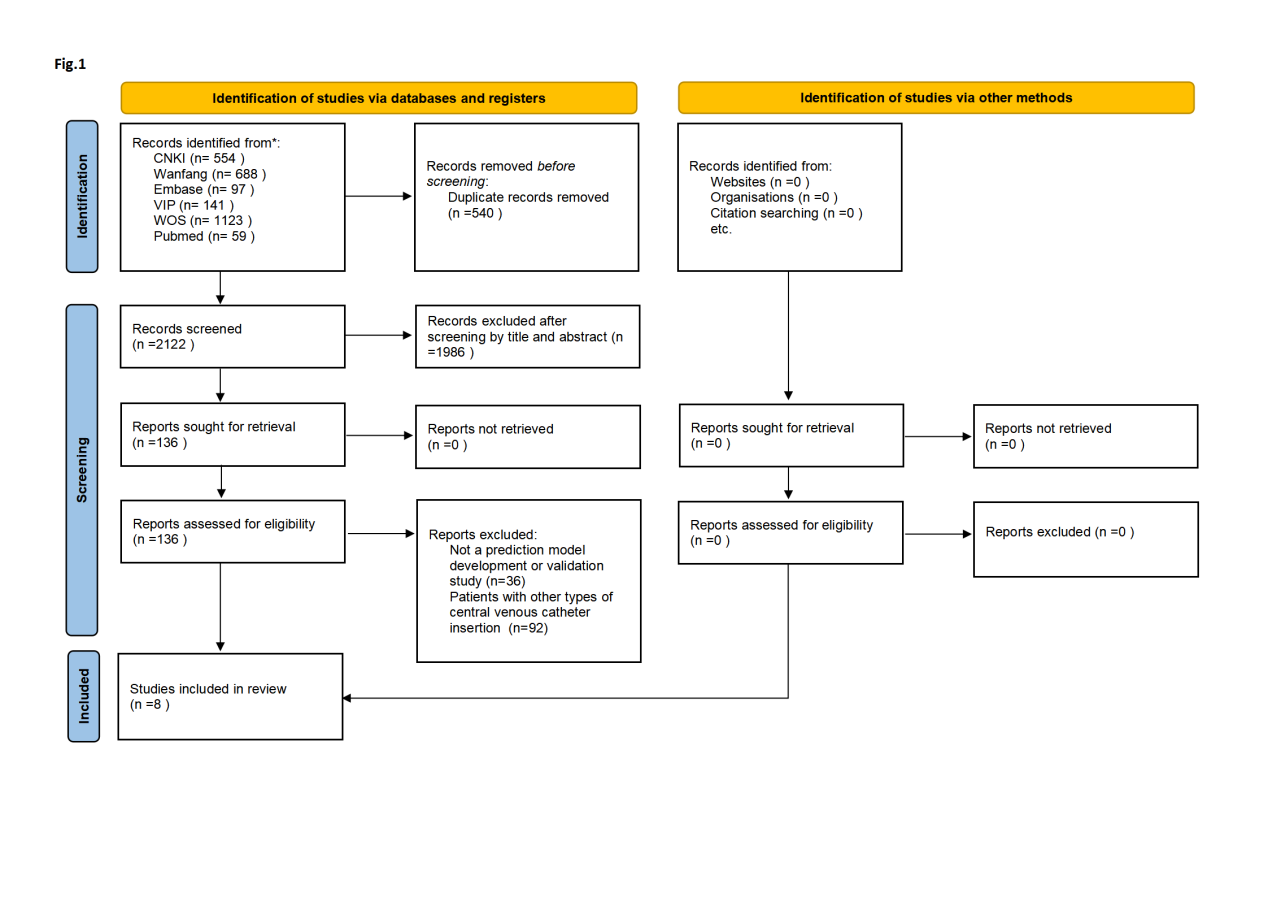
**Fig 1. Flow_diagram.**

**
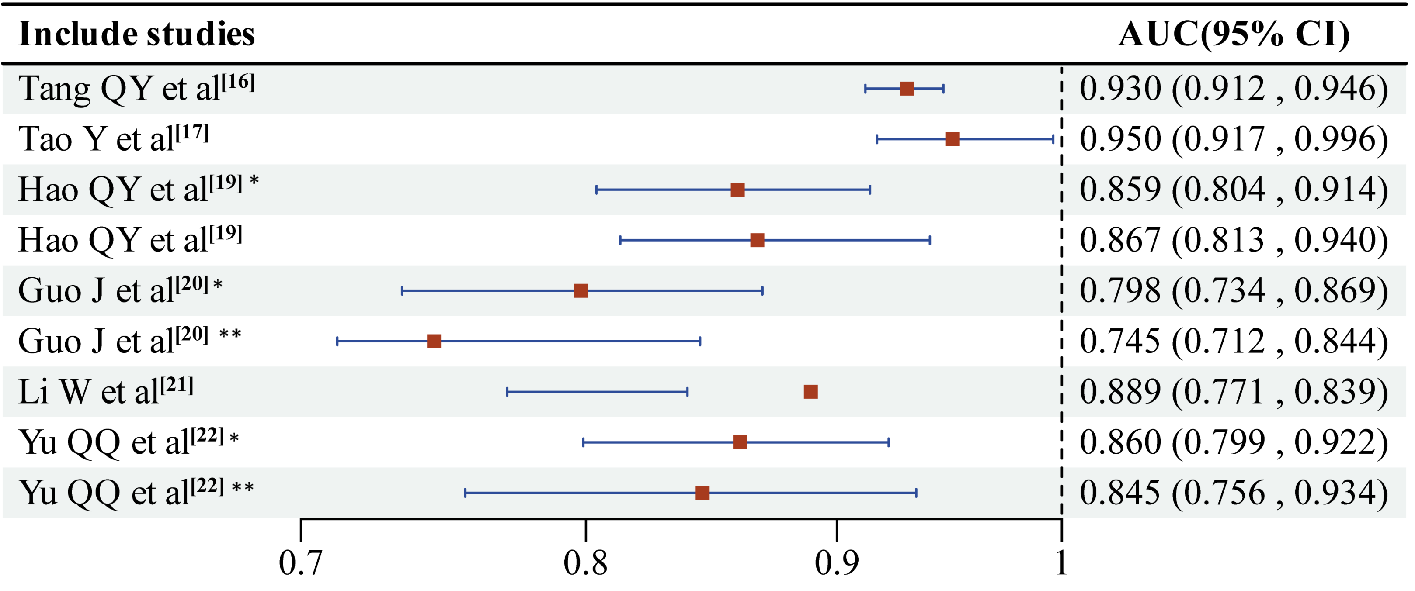
Fig 2. Forest plot of different model performances.** The length of the blue line represents the width of the confidence interval, the red square represents the model's area under the curve (AUC), * represents the development set of the model, and ** represents the validation set of the model.

# Supplemental Tables

**Table 1. Scoping review eligibility criteria.**

| **Inclusion criteria** | **Exclusion criteria** |
| --- | --- |
| Aged ≥ 18 years | Studies including patients with other types of central venous catheterization |
| Received PICC placement | Literature published multiple times |
| Study must focus on the development or validation of a risk model for PICC catheter-related bloodstream infections | Studies discussing only risk factors without model construction |
| The methods for model construction or validation must be clearly described | Literature from which the full text cannot be obtained |
| The literature must be published in Chinese or English. | Conference abstracts and review articles |

**Table 2. Characteristics of the included studies（n=8）**

| **Include studies** | **Publication year** | **Country** | **Research design** | **Study type** | **Subject** | **Sample size** | **CLABSI incidence rate (%)** |
| --- | --- | --- | --- | --- | --- | --- | --- |
| Erica et al^[15]^ | 2017 | America | Multi center prospective cohort study | Development and validation | Hospitalized medical adults | 23088 | 1.10 |
| Tang QY et al^[16]^ | 2020 | China | Retrospective study | Development | Hospitalized adults | 931 | 6.80 |
| Tao Y et al^[17]^ | 2022 | China | Retrospective study | Development and validation | Tumor patients | 11901 | 0.39 |
| Hirotaka et al^[18]^ | 2023 | Japan | Retrospective study | Validation | Hospitalized adults | 1459 | 6.10 |
| Hao QY et al^[19]^ | 2023 | China | Retrospective study | Development and validation | Lung cancer chemotherapy patients | 701 | 10.13 |
| Guo J et al^[20]^ | 2024 | China | Retrospective study | Development and validation | Elderly patients undergoing chemotherapy for acute leukemia | 568 | 8.1 |
| Li W et al^[21]^ | 2024 | China | Retrospective study | Development and validation | Hospitalized adults | 505 | 14.85 |
| Yu QQ et al^[22]^ | 2024 | China | Retrospective study | Development and validation | Tumor patients | 1146 | 0.28 |

**Table 3. Evaluation of bias risk and applicability of included studies**

| **Include studies** | **Risk of bias** | | | | **Applicability** | | | **Overall** | |
| --- | --- | --- | --- | --- | --- | --- | --- | --- | --- |
|  | **Research object** | **Predictor** | **Outcome** | **Analysis** | **Research object** | **Predictor** | **Outcome** | **Bias** | **Applicability** |
| Erica et al^[15]^ | + | + | + | + | + | + | + | + | + |
| Tang QY et al^[16]^ | － | + | + | + | + | + | + | － | + |
| Tao Y et al^[17]^ | － | ? | + | + | + | + | + | － | + |
| Hirotaka et al^[18]^ | + | ? | + | + | + | + | + | － | + |
| Hao QY et al^[19]^ | + | + | + | + | + | + | + | + | + |
| Guo J et al^[20]^ | + | － | + | + | + | + | + | － | + |
| Li W et al^[21]^ | + | ？ | － | － | + | + | + | － | + |
| Yu QQ et al^[22]^ | － | + | + | － | + | + | + | － | + |

**Table 4. Construction and validation of risk prediction model (n=8)**

| **Include studies** | **Modeling method** | **Candidate variables** | **Variable selection method** | **Model predictive factors** | **Model presentation method** | **Missing Data** |
| --- | --- | --- | --- | --- | --- | --- |
| Erica et al^[15]^ | Cox regression | 24 | Cox regression | 6：hematological cancer, CLABSI within 3 months of PICC insertion, multilumen PICC, solid cancers with ongoing chemotherapy, receipt of total parenteral nutrition through the PICC, presence of another central venous catheter (CVC) at the time of PICC placement | Michigan PICC-CLABSI (MPC) score | Multiple imputation |
| Tang QY et al^[16]^ | Logistic regression | 12 | X^2^test, t test, Logistic regression | 8：diabetes mellitus, malignant tumor, hematopathy, parenteral nutrition, double lumen,  additional devices, ICU stay, and the time of indwelling catheter | Nomogram | —— |
| Tao Y et al^[17]^ | Logistic regression | 14 | Logistic regression | 4：Dermatitis, catheter-related thrombosis, local infection and  exudation during PICC retention | Nomogram | —— |
| Hirotaka et al^[18]^ | —— | —— | —— | 6：hematological cancer, CLABSI within 3 months of PICC insertion, multilumen PICC, solid cancers with ongoing chemotherapy, receipt of total parenteral nutrition through the PICC, presence of another central venous catheter (CVC) at the time of PICC placement | Michigan PICC-CLABSI (MPC) score | —— |
| Hao QY et al^[19]^ | Logistic regression | 16 | Logistic regression | 7：Diabetes,  the number of chemotherapy ≥ 5, history of hospitalization in intensive care unit, prolonged catheter maintenance time, catheter movement, catheter retention time ≥ 30 d, and the number of punctures ≥ 2 | Nomogram | —— |
| Guo J et al^[20]^ | Logistic regression | 5 | X^2^ test, Logistic regression | 4：chemotherapy frequency， single catheterization puncture frequency， whether catheterization maintenance frequency was standardized, and catheterization retention time | Nomogram | —— |
| Li W et al^[21]^ | Logistic regression | 13 | LASSO, Logistic regression | 7：age >60 years, catheter movement, catheter maintenance cycle >7 days, direct insertion, poor immune function, complications, body temperature ≥37.2℃ before PICC placement | Nomogram | —— |
| Yu QQ et al^[22]^ | Logistic regressionand extreme learning  Machine (ELM) | 17 | X^2^ test, Logistic regression | 6：diabetes history, number of chemotherapy sessions, maintenance cycle, maintenance address, white blood cell count and albumin | Nomogram, ELM prediction model | —— |

Note: LASSO（the least absolute shrinkage and selection operator regression, LASSO）

**Table 5. Classification of model predictive factors**

| Items | | Number of models included | Items | | Number of models included |
| --- | --- | --- | --- | --- | --- |
| General Information | Age | 1 | Disease related  factor | Dermatitis | 1 |
| Catheter related factors | Multilumen PICC | 3 |  | Infections | 1 |
|  | Attachment | 1 |  | Solid cancers with ongoing chemotherapy | 2 |
|  | Catheterization retention time | 4 |  | Immune function | 1 |
|  | Catheter movement | 2 |  | Body temperature before PICC placement | 1 |
|  | Exudation | 1 |  | Complications | 1 |
|  | Maintenance cycle/frequency | 3 |  | Hematological disease | 1 |
|  | Number of punctures | 2 |  | Malignant tumor | 3 |
|  | Presence of another central venous catheter (CVC) at the time of PICC placement | 2 |  | Diabetes | 3 |
|  |  |  |  | White blood cell count | 1 |
|  |  |  |  | Albumin | 1 |
|  | Direct insertion | 1 | Therapeutic factors | Receipt of total parenteral nutrition through the PICC | 3 |
|  | CLABSI within 3 months of PICC insertion | 2 |  | Chemotherapy frequency | 3 |
|  | Catheter-related thrombosis | 1 | Other factors | ICU hospitalization history | 2 |
|  |  |  |  | Maintenance address | 1 |

**Table 6. Model validation and performance**

| **Include studies** | **Validation** | **AUC^a^/C Index^b^** | **Calibration** | **DCA** |
| --- | --- | --- | --- | --- |
| Erica et al^[15]^ | Internal validation | 0.67 ~0.77^a^ | —— | —— |
| Tang QY et al^[16]^ | Internal validation | 0.930^a^，0.929^b^ | Calibration curve (with an average absolute error of 0.017) | —— |
| Tao Y et al^[17]^ | Internal validation | 0.825^b^ | Calibration curve | —— |
| Hirotaka et al^[18]^ | External validation | —— | The calibration slope is 1.16, P = 0.024, update the calibration slope to 1.02, P = 0.051 | Superior to the original MPC score |
| Hao QY et al^[19]^ | External validation | Modeling set: 0.859^a^  Validation set: 0.876^a^ | Calibration curve: The incidence rate is basically consistent with the actual incidence rate; Hosmer⁃Lemeshow: Modeling set X^2^=8.905, P=0.350； Validation set X^2^=8.693, P=0.365 | —— |
| Guo J et al^[20]^ | External validation | Modeling set: 0.798^a^  Validation set: 0.745^a^ | Calibration curve | Has good clinical application efficacy |
| Li W et al^[21]^ | Internal validation | 0.889^a^ | —— | —— |
| Yu QQ et al^[22]^ | External validation | Nomogram:  Modeling set: 0.860^a^  Validation set: 0.845^a^  ELM prediction model (Modeling set):  R^2^ = 0.823，mean squared error = 0.051 | Hosmer-Lemeshow:  Modeling set X^2^  =5.201，*P*=0.736；Validation set X^2^=6.079，*P*=0.531 | —— |
