# Supplementary material for: Models for predicting the risk of bloodstream infections associated with peripherally inserted central venous catheters: A scoping review
Source: PLoS One. 2025 Oct 6;20(10):e0333466. doi: 10.1371/journal.pone.0333466 (PMC12500127; doi:10.1371/journal.pone.0333466)
Supplement: S3 File — (DOCX) [file pone.0333466.s003.docx]

**Identification of studies via other methods**

**Identification of studies via databases and registers**

Records identified from:

Websites (n =0 )

Organisations (n =0 )

Citation searching (n =0 )

etc.

Records removed *before screening*:

Duplicate records removed (n =540 )

Records identified from*:

CNKI (n= 554 )

Wanfang (n= 688 )

Embase (n= 97 )

VIP (n= 141 )

WOS (n= 1123 )

Pubmed (n= 59 )

**Identification**

Records screened

(n =2122 )

Records excluded after screening by title and abstract (n =1986 )

Reports not retrieved

(n =0 )

Reports sought for retrieval

(n =0 )

Reports sought for retrieval

(n =136 )

Reports not retrieved

(n =0 )

**Screening**

Reports assessed for eligibility

(n =0 )

Reports excluded (n =0 )

Reports assessed for eligibility

(n =136 )

Reports excluded:

Not a prediction model development or validation study (n=36)

Patients with other types of central venous catheter insertion (n=92)

Studies included in review

(n =8 )

**Included**
